# Cartilage endoplasmic reticulum stress may influence the onset but not the progression of experimental osteoarthritis

**DOI:** 10.1186/s13075-019-1988-6

**Published:** 2019-09-11

**Authors:** Louise H. W. Kung, Lorna Mullan, Jamie Soul, Ping Wang, Kazutoshi Mori, John F. Bateman, Michael D. Briggs, Raymond P. Boot-Handford

**Affiliations:** 1 Wellcome Trust Centre for Cell-Matrix Research, Faculty of Biology, Medicine and Health and Manchester Academic Health Science Centre, The University of Manchester, Manchester, M13 9PT UK; 2 Murdoch Children’s Research Institute, Parkville, VIC 3052 Australia; 3 Institute of Genetic Medicine, Newcastle University, International Centre for Life, Central Parkway, Newcastle Upon Tyne, NE1 3BZ UK; 4 Department of Biophysics, Graduate School of Science, and Department of Radiation Genetics, Graduate School of Medicine, Kyoto University, Kyoto, 606-8502 Japan

**Keywords:** Endoplasmic reticulum (ER) stress, Osteoarthritis, Mouse, Medial meniscus destabilisation (DMM), ATF6α, Apoptosis, Histology, RNA-seq

## Abstract

**Background:**

Osteoarthritis has been associated with a plethora of pathological factors and one which has recently emerged is chondrocyte endoplasmic reticulum (ER) stress. ER stress is sensed by key ER-resident stress sensors, one of which is activating transcription factor 6 (ATF6). The purpose of this study is to determine whether increased ER stress plays a role in OA.

**Methods:**

OA was induced in male wild-type (+/+), *ColIITg*^*cog*^ (c/c) and *Atf6α*^*−/−*^ mice by destabilisation of the medial meniscus (DMM). c/c mice have increased ER stress in chondrocytes via the collagen II promoter-driven expression of ER stress-inducing Tg^cog^. Knee joints were scored histologically for OA severity. RNA-seq was performed on laser-micro-dissected RNA from cartilage of +/+ and c/c DMM-operated mice.

**Results:**

In situ hybridisation demonstrated a correlation between the upregulation of ER stress marker, BiP, and early signs of proteoglycan loss and cartilage damage in DMM-operated +/+ mice. Histological analysis revealed a significant reduction in OA severity in c/c mice compared with +/+ at 2 weeks post-DMM. This chondroprotective effect in c/c mice was associated with a higher ambient level of BiP protein prior to DMM and a delay in chondrocyte apoptosis. RNA-seq analysis suggested *Xbp1*-regulated networks to be significantly enriched in c/c mice at 2 weeks post-DMM. Compromising the ER through genetically ablating *Atf6α*, a key ER stress sensor, had no effect on DMM-induced OA severity.

**Conclusion:**

Our studies indicate that an increased capacity to effectively manage increases in ER stress in articular cartilage due either to pre-conditioning as a result of prior exposure to ER stress or to genetic pre-disposition may be beneficial in delaying the onset of OA, but once established, ER stress plays no significant role in disease progression.

**Electronic supplementary material:**

The online version of this article (10.1186/s13075-019-1988-6) contains supplementary material, which is available to authorized users.

## Background

Osteoarthritis (OA) is a debilitating degenerative joint disease with risk factors ranging from gender, genetic pre-disposition, obesity, injury, joint alignment and age [1–3]. This multitude of risk factors, along with variable clinical features, emphasises OA heterogeneity [4, 5]. The pathological hallmarks of OA include progressive fibrillation of articular cartilage, altered chondrocyte phenotype, thickening of subchondral bone and remodelling of peri-articular tissues [6]. The characteristic progressive breakdown of cartilage in OA joints results from an imbalance between the degradation and repair of the tissue, and one potential pathogenic mechanism that has received recent attention is increased chondrocyte endoplasmic reticulum (ER) stress.

ER stress can be caused by many different factors and triggers the unfolded protein response (UPR) [7–10]. The UPR is mediated by three ER-resident transmembrane stress sensors: pancreatic ER-kinase (PKR)-like ER kinase (PERK), inositol-requiring enzyme 1 (IRE1) and activating transcription factor 6 (ATF6), and results in attenuation of protein translation and upregulation of chaperones (including BiP/Grp78) and genes involved in ER-associated degradation. The transcriptional upregulation of BiP has been used extensively as a marker of ER stress [11, 12]. The initial responses to ER stress are aimed at alleviating the stress although should these responses fail to restore protein homeostasis, CHOP-mediated apoptosis may be triggered [13].

Chondrocytes, due to their high secretory load, are particularly sensitive to ER stress [10]. Indeed, elevated chondrocyte ER stress is not only associated with a number of chondrodysplasias but represents the principal disease mechanism in at least one of these forms of dwarfism [10, 14–16]. Over recent years, several studies have associated ER stress with OA in both man and mouse [17–22]. In all cases, an upregulation of the ER chaperone BiP was reported in osteoarthritic tissue. However, it remains unclear whether increased ER stress is merely a physiological response, for instance to the anabolic, increased ECM synthesis seen in OA, or it can influence the onset and progression of disease.

Our present study was therefore to determine whether exposure to increased ER stress can modulate OA onset or progression. We previously generated a transgenic mouse model (*ColIITg*^*cog*^; [23]) in which ER stress was induced in chondrocytes by the *Col2a1* promoter-driven expression of a misfolding protein, the *cog* form of thyroglobulin (Tg^cog^), in a similar fashion to that described previously [15]. Briefly, Tg^cog^ misfolds in the ER inducing ER stress and an UPR [14, 23, 24]. We also investigated a possible connection between disrupting the cells ability to sense ER stress and disease progression through ablating one of the key ER stress sensors ATF6α. *Atf6α*^−/−^ mice have no overt phenotype; however, their ability to respond to ER stress challenges, including through the induction of BiP, is severely compromised [25]. We induced OA by destabilisation of the medial meniscus (DMM) in *ColIITg*^*cog*^ and *Atf6α*-knockout mice [25] to examine the effects of either prior exposure to increased ER stress or a compromised UPR on disease onset and progression, respectively. Our results show increased ER stress is an early response in surgically induced OA. The *ColIITg*^*cog*^ mice, whose chondrocytes have been pre-conditioned to increased ER stress throughout development [23], are protected against the earliest stages of OA. Furthermore, *Atf6α*-knockout mice, which have a diminished ability to ameliorate ER stress, showed no significant differences in OA severity compared with control mice, suggesting ER stress is not a significant factor in disease progression.

## Methods

### Mouse strains

Generation of the *ColIITg*^*cog*^ mouse has been previously described [23]. Mice were bred to homozygosity, and breedings of wild-type (+/+) and homozygote (c/c) mice were maintained on a FVB/N/C57Bl6 background. *Atf6α*^*−/−*^-knockout mice were generated as described previously [25].

### Surgical induction of osteoarthritis

OA was induced in 10–12-week-old male mice by DMM of the right knee as described [26]. Briefly, under isoflurane anaesthesia and following standard surgical site preparation, the medial menisco-tibial ligament was exposed by gentle blunt dissection and transected with a 5-mm micro-surgical blade using sterile surgical techniques. Joints were flushed with sterile saline prior to separate closure of the joint capsule, subcutaneous tissue (8/0 polyglactin 910 suture) and skin (cyanoacrylate). The left knee was the non-operated control. Operated mice received post-operative pain relief (buprenorphine) for 48 h. For RNA-seq analysis, sham operations where the medial menisco-tibial ligament was visualised but not transected were performed in independent animals. Mice were randomly allocated to groups/time points prior to study commencement using their individual ID numbers.

### Histology

Animals were sacrificed at 2 (*n* = 6/group), 4 (*n* = 13/group) and 8 (*n* = 14/group) weeks post-surgery. Knee joints were dissected, fixed in paraformaldehyde overnight, demineralised using 0.8 M EDTA pH 7.4 for 1–2 weeks, paraffin-embedded, sectioned coronally at 5 μm thickness, stained with safranin O and fast-green and photographed as previously described [23].

OA severity was determined using the Osteoarthritis Research Society International (OARSI) scoring system [27]. At least five sections across the whole joint, spaced ~ 80 μm apart, were scored by three separate scorers in a blind fashion. The joint score was calculated by combining the individual scores from all four quadrants of the joint. Statistical differences between groups were evaluated using ANOVA (GraphPad Prism).

### Immunohistochemistry (IHC)

IHC was performed as described [23]. Briefly, antigen unmasking was carried out in citrate buffer pH 6.0 heated to > 85 °C for 10 min followed by washes in PBS. Primary antibody used was anti-Grp 78 goat polyclonal (Santa Cruz, SC-1051) diluted 1/300. Sections were then incubated with ABC reagent (Vector Laboratories, PK-6100) for 30 min and developed using the Vector VIP kit (Vector Laboratories, SK-4600). Negative control sections for IHC were performed with the appropriate serum minus the primary antibody. Tissue sections that were known to express the protein of interest were included as positive controls. Images were captured using the Carl Zeiss Axiovision microscope fitted with an Axiocam colour CCD camera and associated Axiovision software.

### In situ hybridisation (ISH)

DIG-labelled colourimetric in situ was performed as described [15] with the following riboprobes: Col2a1: 600 bp insert encoding the 3′UTR from I.M.A.G.E clone ID 735113; BiP: 350 bp fragment from I.M.A.G.E clone ID6334883; thyroglobulin: 700 bp cDNA fragment amplified using primers F:TTGTAGATCCATCCATCAAGC and R:GTGACTACGATGAAGTTGC.

### Terminal transferase dUTP nick end labelling (TUNEL) assay

TUNEL assays were performed using a fluorometric TUNEL kit (Promega, G3250) following the manufacturer’s instructions. Images were collected using the CoolSNAP ES Olympus BX51 camera and associated Metaview software. Two slides containing sections spaced at least 80 μm apart, per animal, were assayed. Separate cell counts throughout the entire lateral and medial tibial plateau were assessed as described previously [23] and analysed by ANOVA (GraphPad Prism).

### Laser capture microdissection of articular cartilage, RNA preparation and RNA sequencing

Dissection, laser microdissection and RNA extraction from mouse articular cartilage were performed as previously described [28, 29]. Each mouse articular cartilage yielded ~ 20–30 ng of total RNA. +/+ SHAM, +/+ DMM and c/c DMM mice at 2 weeks post-surgery (*n* = 3/group) were used in this experiment. The integrity of total RNA was assessed on the Agilent Technology Bioanalyzer 2100 using RNA6000 Pico-Chip (Agilent) and quantified using a QuBit assay (Life Technologies). RNA sequencing cDNA libraries were generated using the NuGen Ovation® Single Cell RNA-Seq system (NuGEN Technologies Inc., Part No.0342) according to the manufacturer’s instructions. Adapter indices were used to multiplex libraries, which were pooled prior to cluster generation using a cBot instrument. The loaded flow-cell was then paired-end sequenced (100 + 100 cycles, plus indices) on an Illumina HiSeq2500 instrument. Demultiplexing of the output data (allowing one mismatch) and BCL-to-Fastq conversion was performed with CASAVA1.8.3. The read counts for each sample (at least 10 M) were normalised to the total number of reads and analysed using DESeq2 at a 10% FDR (adjusted for multiple testing using the Benjamini-Hochberg correction: *p*_adj_ value reported by DESeq). Differentially expressed genes were used in pathway and functional analyses (Ingenuity Systems).

### Quantitative polymerase chain reaction (qPCR)

Due to the limited amounts of tissue RNA available, cDNA libraries from the same pools used for RNA sequencing analysis were used for qPCR. Reactions were performed in duplicate using the SYBR Green kit (Applied Biosystems) following the manufacturer’s instructions on a StepOne Plus detector system (Life Technologies). Primer sequences (Additional file 1: Table S1) used had been previously validated [29] or were designed for this study using Primer-Blast. Relative gene expression levels were normalised to β-actin and calculated using the 2^−ΔCt^ method. To calculate fold changes of gene expression, the 2^−ΔΔCt^ method was used.

## Results

### DMM increases ER stress in cartilage as OA damage becomes apparent

**Fig. 1:**
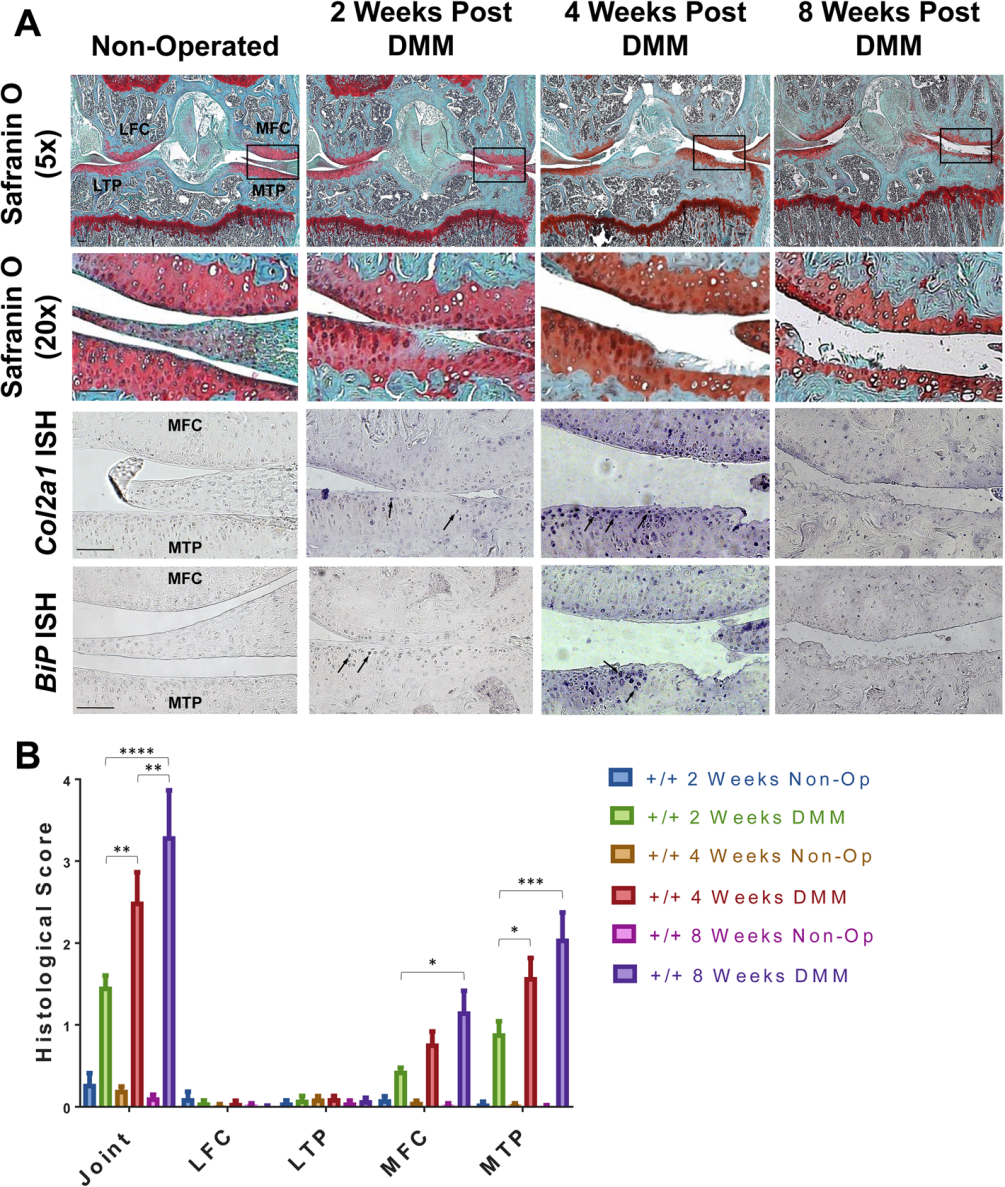
Surgical induction of OA in +/+ mice causes increased ER stress in articular chondrocytes. **a** Safranin O staining of sections from non-operated and DMM-operated knee joints from +/+ mice at 2, 4 and 8 weeks post-DMM. The insert shows an expanded view of the highlighted black box. +/+ mice developed progressive OA as evidenced by the loss of safranin O staining and increasing degree of cartilage degradation. Col2a1 and BiP in situ hydrisation (ISH) was performed on sections from non-operated control joints and DMM-operated joints at 2, 4 and 8 weeks post-DMM. The presence of transcript is indicated by the blue staining (arrows). +/+ DMM-operated knee joints exhibited an increase in Col2a1 and BiP expression compared to non-operated joints. **b** Histological scores of OA pathology using a semi-quantitative scoring system. +/+ mice exhibited significant increase in the severity of OA with time. Results are the average score ± SEM. *p < 0.05, **p < 0.01, ***p < 0.001, ****p < 0.0001. LFC = lateral femoral condyle; LTP = lateral tibial plateau; MFC = medial femoral condyle; MTP = medial tibial plateau; Non-op = non-operated. Scale bar = 100 μm

Lateral compartments of DMM joints and non-operated control joints from +/+ mice showed no significant OA-related changes (Fig. 1). DMM-operated +/+ mice developed OA progressively over the 8-week period with cartilage damage specifically localised to the medial compartments of the joint (Fig. 1a, b). Two weeks post-DMM, +/+ mice exhibited focal loss of proteoglycans and mild fibrillation at the articular surface. By 4 weeks post-DMM, there was cartilage structural damage and vertical cleft formation. At 8 weeks, DMM joints exhibited a significant degree of cartilage degradation. OARSI scoring revealed a significant increase in the severity of OA with time (Fig. 1b).

mRNA expression of *Col2a1* and the ER stress marker *BiP* in articular cartilage of +/+ non-operated knee joints was below the level of detection by ISH (Fig. 1a). In comparison, the +/+ DMM-operated knees exhibited increases in *Col2a1* and *BiP* expression that were evident at 2 weeks and most intense at 4 weeks post-DMM. The spatial localisation of increased expression of both *BiP* and *Col2a1* expanded in tandem with the progression of cartilage damage, especially evident between 2 and 4 weeks post-DMM. The most intense upregulation of expression localised to chondrocytes immediately adjacent to the OA lesion, particularly apparent at 4 weeks post-DMM (Fig. 1a). The upregulation of *BiP* mRNA indicates that increased ER stress is an early feature of disease onset in this mechanically induced OA and is coincidental with first signs of proteoglycan loss and structural damage.

### *ColIITg*^*cog*^ mice are protected against the initial stages of surgically induced OA

To determine whether prior exposure to increased ER stress can influence the onset or progression of OA, we conducted DMM surgery on *ColIITg*^*cog*^ (c/c) mice which experience chondrocyte ER stress when the mutant thyroglobulin transgene, driven by the *Col2a1* promoter, is expressed [23]. We hypothesised that following DMM surgery, the added increased ER stress experienced by chondrocytes as they upregulated collagen II (and therefore Tg^cog^) synthesis would increase either the rate of onset or the severity of disease in c/c mice.

**Fig. 2:**
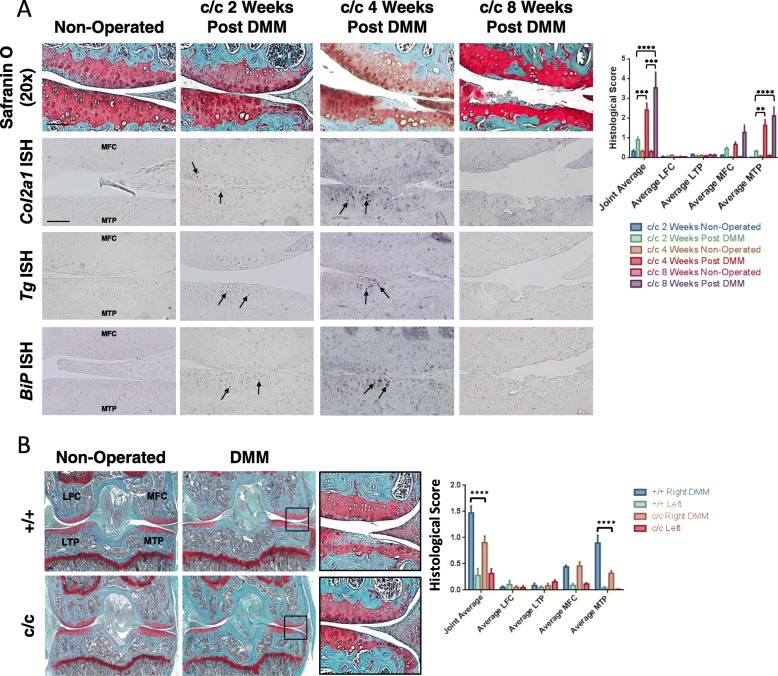
*ColIITg*^*cog*^ mice are protected against the initial stages of surgically induced OA. **a** Safranin O staining and in situ hybridisation (ISH) for *Col2a1*, *Tg*^*cog*^ and *BiP* mRNA on sections from non-operated and DMM-operated knee joints from c/c mice at 2, 4 and 8 weeks post-DMM. The presence of each transcript is indicated by the blue staining and depicted by the arrows. c/c DMM-operated knee joints exhibited an upregulation in all three transcripts, *Col2a1*, *Tg*^*cog*^ and *BiP* in chondrocytes around the OA-affected region compared to c/c non-operated joints. Histological scores of the SO-stained images using a semi-quantitative scoring system are shown. c/c mice exhibited a significant increase in the severity of OA with time. **p < 0.01, ***p < 0.001, ****p < 0.0001. **b** Safranin O staining of sections from non-operated and DMM-operated knee joints from +/+ and c/c mice at 2 weeks post-DMM. The insert shows an expanded view of the highlighted black box. Histological scores of the safranin O-stained images using a semi-quantitative scoring system are shown. c/c mice show significantly reduced OA severity compared with +/+ mice. Data shown as average score ± SEM. ****p < 0.0001 +/+ 2 week post-DMM versus c/c 2 week post-DMM. LFC = lateral femoral condyle; LTP = lateral tibial plateau; MFC = medial femoral condyle; MTP = medial tibial plateau. Scale bar = 100 μm

We first established that the expression of the *ColIITg*^*cog*^ transgene per se during development and early adulthood did not predispose the animals to OA or other articular cartilage abnormalities (Additional file 2: Figure S1A&B). The articular cartilage of *ColIITg*^*cog*^ mice at 3–9 weeks and 18 months of age were normal with no signs of degeneration and were indistinguishable from +/+ mice (Additional file 2: Figure S1A&B). Next, DMM surgery was conducted on c/c mice as part of the same experiment that generated the +/+ (control) data described above (Fig. 1). It should be noted that in the cartilage of non-operated knees in c/c mice, the expression of *Col2a1*, *Tg*^*cog*^ and *BiP* was below the level of detection (Fig. 2a). The lack of detectable expression demonstrates that in an unchallenged adult knee joint *Col2a1* expression (and thus *Tg*^*cog*^ and *BiP*) is very low in contrast to the high levels seen in younger animals. Increased ER stress indicated by the upregulation of *BiP* mRNA accompanying *Tg*^*cog*^ and *Col2a1* expression was apparent in chondrocytes following DMM surgery in c/c mice compared to non-operated c/c controls (Fig. 2a). Furthermore, DMM-operated c/c mice developed signs of osteoarthritic damage (Fig. 2a) in a similar fashion to the controls (+/+) described above (Fig. 1). However, direct comparison of the level and type of damage revealed that at 2 weeks post-DMM, c/c mice had significantly less damage than the +/+ controls (Fig. 2b). The loss of proteoglycan and cartilage damage in the medial tibial plateau was significantly less in c/c mouse joints (Fig. 2b). However, at later time points there were no differences in the levels of damage seen in the two groups (cf histological scores for 4 and 8 weeks +/+ (Fig. 1) and c/c mice (Fig. 2)).

### Apoptosis is delayed in DMM-operated *ColIITg*^*cog*^ mice compared with +/+ mice

**Fig. 3:**
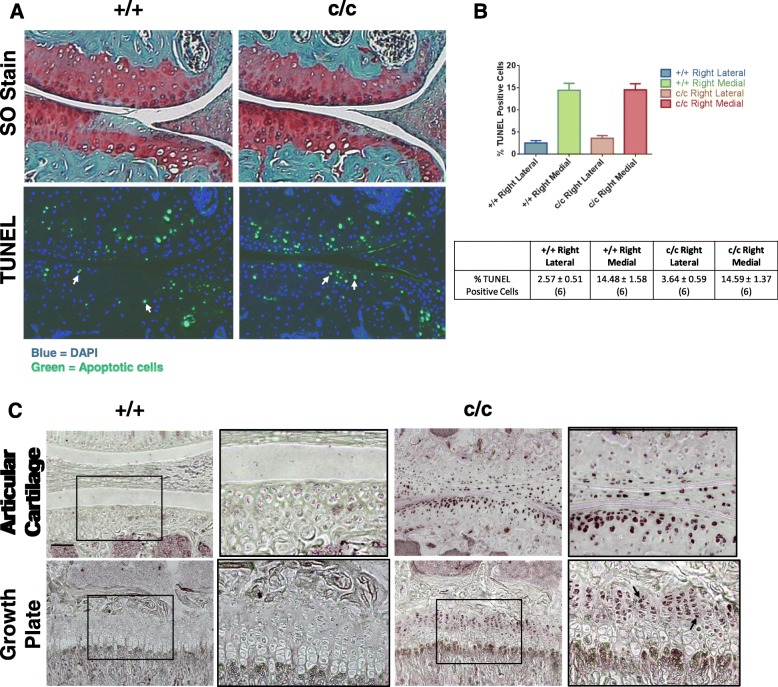
Chondrocyte apoptosis associated with proteoglycan loss is delayed in DMM-operated *ColIITg*^*cog*^ mice which exhibit increased levels of intracellular BiP protein prior to DMM. Apoptosis is delayed in mice homozygous for *ColIITg*^*cog*^ (c/c) compared to wild-type (+/+) mice. **a** Representative knee joint medial compartment sections stained with safranin O (SO) to visualise the OA lesion at 2 weeks DMM and serial sections analysed by TUNEL (FITC) with DAPI counter-stain (blue). **b** Quantitation of TUNEL-positive cells shows a significant increase in apoptosis in the medial compartments of the 2-week DMM-operated joints compared to the lateral compartments in both +/+ and c/c mice (see **a**). Chondrocytes within the OA lesion in +/+ mice do not stain with DAPI, whereas chondrocytes in the OA lesion of c/c mice are labelled with TUNEL and DAPI (see arrows) indicating they are in the process of apoptosis and that they are delayed compared with +/+ mice. **c** Increased BiP protein (dark purple staining indicated by black arrows) in articular and growth plate chondrocytes of 9-week-old non-operated c/c compared to +/+ mice. The inserts are enlarged sections of the areas represented by the black boxes. Scale bar = 100 μm

To investigate the mechanism by which the *ColIITg*^*cog*^ transgene appeared to have a chondroprotective role during the onset of OA (2 weeks DMM), we examined the levels of apoptosis in the cartilage of +/+ and c/c mice following DMM (Fig. 3a). The lateral compartments of both +/+ and c/c mice 2 weeks post-DMM exhibited few apoptotic cells (Fig. 3b). In comparison, significant numbers of apoptotic cells were detected in the medial compartments of DMM-operated joints (Fig. 3a) although there was no significant difference in the proportion of apoptotic cells in +/+ and c/c cartilage (Fig. 3b). Nevertheless, close inspection of the OA lesion revealed a subtle but clear delay in apoptosis in 2-week DMM-operated joints of c/c compared to +/+ mice. Chondrocytes within the OA lesion of +/+ mice did not stain with DAPI (blue), nor were they labelled as apoptotic (green), indicating that these cells were already dead and their genomic DNA degraded (Fig. 3a). It was the chondrocytes immediately adjacent to the OA lesion that were TUNEL-positive in the +/+ medial compartment (Fig. 3a—see arrows). However, in c/c mice, the cells within the lesion were TUNEL-positive, indicating that apoptosis in these cells was still in progress and therefore delayed compared to the equivalent cells in +/+ controls (Fig. 3a).

### *ColIITg*^*cog*^ mice articular chondrocytes exhibit a higher level of BiP protein prior to DMM surgery

As described above for both +/+ and c/c mice, *BiP* mRNA levels in chondrocytes of non-operated joints were below the level of detection by in situ analyses and only became detectable in OA-affected chondrocytes 2 weeks post-DMM. We next looked to see whether BiP protein levels in the cartilage prior to DMM induction were equivalent in +/+ and c/c mice (Fig. 3c). Surprisingly, immunodetectable BiP was much more intense in chondrocytes of c/c mice compared to +/+ mice even though in both lines, mRNA levels for *BiP* were very low (Figs. 1 and 2). This increase in BiP protein was still apparent in the c/c mouse cartilage 2 weeks after DMM (Additional file 2: Figure S2). These data indicate that chondrocytes in the *ColIITg*^*cog*^ line have a higher ambient concentration of BiP protein, presumably due to the prior exposure to increased levels of ER stress during development [23]. The increased concentration of BiP chaperone and the implicit increased capability to neutralise the damaging effects of elevated ER stress could explain why the onset of OA and chondrocyte apoptosis was slightly delayed in the c/c compared to +/+ line.

### RNA sequencing analysis identifies dysregulated genes 2 weeks after DMM surgery

To investigate the gene expression changes associated with the chondroprotective effects in the c/c mice, we performed RNA sequencing on articular cartilage from 2 weeks DMM and SHAM knee joints. The expression profiles of 6 genes were assessed by PCR and compared with the RNA-seq data in the 3 groups to validate the expression data (Additional file 2: Figure S3). A total of 511 dysregulated genes were identified in +/+ DMM versus SHAM (Additional file 3: Table S2) and 2090 in c/c DMM versus SHAM (Additional file 3: Table S3). Comparing c/c DMM vs +/+ DMM revealed 633 genes that were either up- or downregulated (Additional file 3: Table S4).

The 50 genes most significantly dysregulated by DMM in +/+ mice were compared with the equivalent expression profiles in c/c DMM mice (Tables 1 and 2). Expression of 49 of the 50 genes were detected in c/c DMM mice. All but one of these 49 genes changed expression in the same direction in both +/+ and c/c mice; 39 of which were also statistically dysregulated in c/c DMM cartilage (*p* < 0.05; Table 1). In addition, the genes indicated by an asterisk (Table 1) were also found to be differentially regulated in a concordant fashion in an independent microarray analysis of 2-week DMM-induced changes [29]. Ingenuity analysis suggested the upstream regulators of differentially expressed genes in +/+ DMM cartilage compared with SHAM to include TGFβ1 & 3, EGF, FGF2 and TNF (Additional file 4: Table S5).

**Table 1:** Fifty most dysregulated genes in articular cartilage of +/+ DMM compared to SHAM and the equivalent expression in the *ColIITg*^*cog*^ DMM group

WT DMM v SHAM			*ColIITg cog* DMM v SHAM	
*p*_adj_	Fold change	Gene name	Fold change	*p*_adj_
3.91E−63	11.51	Inhba*	32.40	2.13E−102
1.0308E−29	−8.60	Adamts18*	− 3.93	5.81E−08
2.85E−27	6.85	Bmp7*	6.93	3.89E−24
1.1299E−24	4.33	*Gpx3*	7.45	8.48E−36
9.0276E−24	4.23	*Pappa*	6.26	4.43E−13
5.1868E−21	3.30	Fn1*	5.35	6.87E−22
7.9751E−21	3.74	Ank*	4.51	1.70E−27
2.4681E−20	3.52	Ctsh*	3.05	2.73E−06
7.7114E−19	5.44	Penk*	8.26	4.71E−27
4.0057E−18	5.00	Prkg2*	5.09	6.04E−13
5.266E−18	−3.87	Gas1	− 1.01	NS
7.7024E−18	−2.86	*Fibin*	− 1.82	1.03E−06
3.6914E−17	−3.07	Scara3*	− 1.30	NS
8.5273E−17	5.33	Hbegf*	8.52	1.14E−17
4.1624E−15	2.47	*Pmepa1*	3.65	9.44E−31
4.6948E−15	2.43	Tm4sf1*	4.13	3.90E−46
4.7632E−15	2.38	Galnt1*	2.90	3.33E−21
8.6454E−15	2.91	Hspa4l*	4.20	2.41E−38
1.9886E−14	−2.39	*Comp*	− 1.42	0.04857489
5.7638E−14	2.19	Col6a3*	2.56	2.20E−10
9.9399E−14	3.50	Bmp2*	4.52	2.87E−12
9.9399E−14	2.88	*Mgp*	10.33	1.91E−176
6.1568E−13	−6.41	Adamtsl2*	−3.04	0.020992517
1.585E−12	3.93	Npr3*	2.82	0.00016205
2.4475E−12	2.99	Fgf1*	1.32	NS
5.4612E−12	3.32	Bhlhe40*	4.46	3.58E−18
1.0334E−11	−3.05	Chad*	− 1.68	0.000147026
2.5909E−11	3.29	*Serpine1*	3.47	3.56E−07
3.7608E−11	3.47	Serpina3n*	22.32	NA
8.3862E−11	−3.15	*Abi3bp*	− 2.12	8.81E−08
8.3862E−11	−3.79	*Ppp1r3c*	− 2.52	0.000331801
2.0827E−10	5.13	*Gcnt4*	5.37	1.97E−08
2.3682E−10	−6.84	Omd*	− 2.47	0.0655615
2.5468E−10	2.36	Uaca*	2.84	3.35E−31
5.2059E−10	3.88	Fhl2*	3.45	1.43E−08
6.9381E−10	5.09	Ltbp2*	7.81	NA
6.9381E−10	3.34	Cyr61*	3.39	1.54E−07
3.0948E−09	7.13	Ptgs2*	15.18	NA
4.7855E−09	2.42	*Cplx2*	1.42	0.03383883
5.5902E−09	−2.03	Cytl1	− 1.04	NS
6.2564E−09	1.76	Atp1a1*	2.44	4.53E−22
7.6081E−09	2.50	Tsc22d1*	3.35	1.32E−14
1.598E−08	−2.44	*Gm16070*	− 1.66	0.017891341
2.3008E−08	−1.92	Lox	1.29	0.019344488
3.2292E−08	1.85	Col6a2*	2.73	2.23E−08
4.9035E−08	1.91	*Lmo4*	1.36	0.037644471
6.6389E−08	4.58	Hmga2*	6.95	3.62E−11
8.9945E−08	2.35	Emp1*	3.26	5.00E−09
1.312E−07	6.00	Gdf6	ND	NA
1.52E−07	−2.07	Nampt	− 1.15	NS

Data compiled from Additional file 2: Table S2 &S3. Italics—genes significantly dysregulated in both WT DMM and *ColIITg*^*cog*^ DMM compared to SHAM. *Gene also significantly dysregulated in similar direction in 2-week post-DMM wild-type cartilage samples assessed by microarray [29]. *ND* not detected, *NA* not available due to only one value in data set, *NS* not significant (false discovery rate/adjusted *P* value > 0.1)

**Table 2:** Fifty most significantly dysregulated genes in articular cartilage of *ColIITg*^*cog*^ DMM compared to +/+ DMM mice

Gene name	Fold change	*p*_adj_
Enpp2	3.80	7.729E−24
Apobr	− 5.38	1.581E−18
Gas1	3.90	1.581E−18
Serpina3n	5.76	3.413E−16
Chil1	3.32	9.585E−15
Ltf	− 5.29	1.778E−14
Ighg2b	− 13.55	4.240E−13
Fam111a	− 5.01	5.242E−13
Tnfrsf11b	2.07	1.495E−12
Lyz2	− 3.05	2.941E−12
Mgp	3.22	3.442E−12
Itga2b	− 7.20	1.080E−11
Lox	2.43	7.576E−11
Cpq	2.78	1.069E−10
Eno2	6.51	2.247E−10
Aph1b	− 9.03	8.851E−10
Scara3	2.37	1.834E−09
Camp	− 4.11	2.179E−09
Rcan1	3.15	5.282E−09
Alox5	− 9.00	6.821E−09
Sema3c	3.29	1.006E−08
Pabpc1	− 1.83	2.472E−08
Pcsk5	2.35	2.829E−08
Hells	− 5.99	6.300E−08
Hbb-bt	− 4.42	1.544E−07
2810474O19Rik	− 2.22	1.727E−07
Magt1	2.26	2.385E−07
Gda	− 3.70	2.393E−07
S100a8	− 3.75	4.021E−07
Plekha2	− 2.69	4.102E−07
Clmp	2.27	4.102E−07
Pkd2	2.19	4.102E−07
Dcn	2.07	4.102E−07
Ank2	2.29	1.365E−06
Fkbp9	1.84	1.590E−06
Rps2-ps13	− 6.65	1.822E−06
Tm4sf1	1.65	2.902E−06
Vill	− 5.60	3.656E−06
Ncf1	− 3.48	3.656E−06
Cenpf	− 3.95	4.238E−06
Mcfd2	2.69	4.270E−06
Ccdc125	− 6.25	4.462E−06
Spp1	2.95	4.873E−06
Celf2	− 3.53	5.489E−06
Serpina3g	5.40	5.552E−06
Aqp1	− 2.45	5.886E−06
Lta4h	− 3.62	6.559E−06
Mki67	− 3.47	8.515E−06
Hist1h2ai	− 2.85	8.954E−06
Tmsb4x	− 2.51	9.833E−06

Ingenuity-generated upstream regulator analyses of the full list of differential genes suggested CDKN2A (a cell cycle regulatory gene), Xbp1 (a transcription factor governing key aspects of the UPR), Rictor (a regulatory element of MTORC2) and TGFB1 as being potentially involved in producing the chondroprotective role seen in c/c mice at 2 weeks post-DMM (Table 3).

**Table 3:**
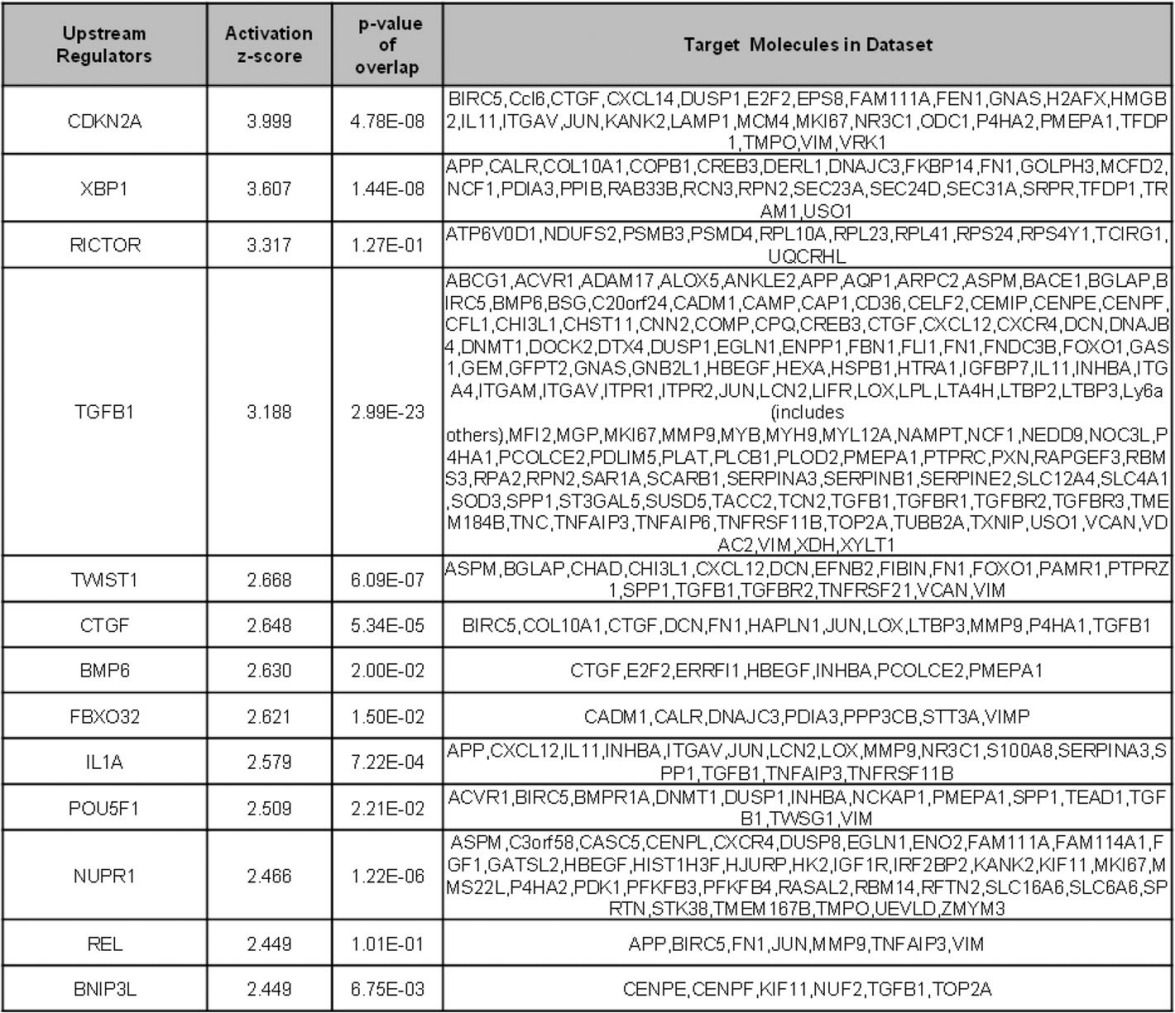
Top upstream regulators identified in *ColIITg*^*cog*^ DMM mice compared to +/+ DMM mice

### Compromising the ATF6α arm of the UPR does not alter the severity of OA

Given that we (Fig. 1) and others have shown that chondrocyte ER stress is a feature of cartilage damage in OA, we were surprised to find that the *ColIITg*^*cog*^ mice did not develop more severe damage following DMM compared to controls. We therefore decided to probe the potential link between increased ER stress and OA disease in a different setting in which the capacity of cells to manage increases in ER stress is compromised due to the ablation of a key ER stress sensor. ATF6α is one of the three key ER stress sensors, and although *Atf6α*^−/−^ mice have no overt phenotype, their ability to respond to ER stress challenges is compromised [25]. For instance, the chondrocytes’ ability to induce BiP and CHOP in response to ER stress caused by the expression of mutant type X collagen in the metaphyseal chondrodysplasia-type Schmid mouse is much impaired and the clinical phenotype more severe on the *Atf6α*^*−/−*^ background [30].

**Fig. 4:**
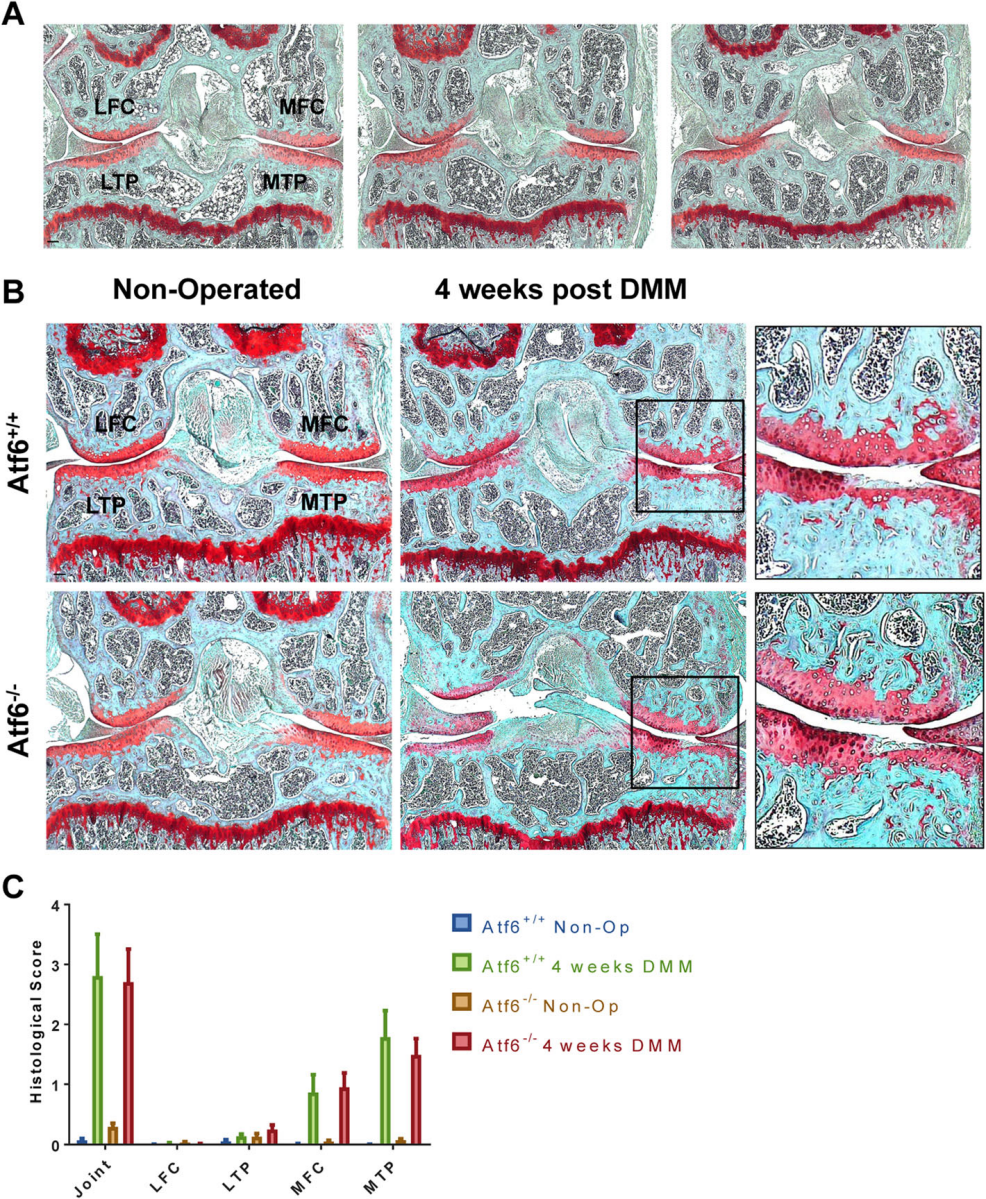
ATF6-knockout mice have normal articular cartilage and develop OA following DMM at the same rate as wild-type controls. **a** Representative images of safranin O-stained sections from knee joints of 6–7-month-old ATF6α-knockout mice (*n* = 3) showing intact articular cartilage with no signs of degeneration. **b** Safranin O-stained sections of non-operated (left) and DMM-operated (right) knee joints from wild-type (WT; *n* = 7) and ATF6α KO (KO; *n* = 9) mice at 4 weeks post-DMM. The insert shows an expanded view of the highlighted black box. **c** Histological scores of the safranin-stained sections. Values are mean ± SEM (*n* = 7). LFC = lateral femoral condyle; LTP = lateral tibial plateau; MFC = medial femoral condyle; MTP = medial tibial plateau. Scale bar = 100 μm

Firstly, histological examination of the articular cartilage of 6–7-month-old *Atf6α*^−/−^ mice revealed no signs of degeneration (Fig. 4a) indicating that these mice do not spontaneously develop OA. We performed DMM surgery on *Atf6α*^−/−^ and *Atf6α*^+/+^ controls at 10–12 weeks of age and collected knee joints 4 weeks later which is the usual first time point examined in this type of study. Non-operated knee joints from *Atf6α*^−/−^ and *Atf6α*^+/+^ mice showed no signs of cartilage degradation as expected (Fig. 4b). In contrast, DMM joints from *Atf6α*^−/−^ and *Atf6α*^+/+^ mice displayed signs of proteoglycan and cartilage loss (Fig. 4b). Histological scoring revealed no differences in the severity of OA between *Atf6α*^−/−^ and *Atf6α*^+/+^ mice (Fig. 4c). The lack of significant differences in the pathology between *Atf6α*^−/−^ and *Atf6α*^+/+^ mice suggests ER stress does not play an important role in DMM-induced OA pathology.

## Discussion

We aimed to establish the role of ER stress in modulating OA disease onset and progression. Firstly, we demonstrated that increased ER stress, indicated by elevated *BiP* mRNA, is a feature of disease onset in the DMM model, being present in chondrocytes at or adjacent to the initial site of damage (Fig. 1a). Cells expressing increased *BiP* had also upregulated *Col2a1* mRNA which indicates these chondrocytes were undergoing an anabolic response to DMM surgery (Fig. 1a). The association of increased BiP with increased collagen synthesis in human OA was previously noted by Nugent et al. [18].

We next examined whether exposure to ER stress is a critical factor in OA onset or progression using the *ColIITg*^*cog*^ (c/c) transgenic mouse [23]. ISH revealed undetectable levels of *Col2a1* expression in the cartilage of unchallenged, non-operated joints of both +/+ and c/c adult mice (Figs. 1 and 2) demonstrating that the collagen II-driven expression of Tg^cog^ which was high in actively growing animals [23] was not expressed at significant levels in adult *ColIITg*^*cog*^ mice. Indeed, *Tg*^*cog*^ mRNA was not detected by ISH in articular cartilage of non-operated joints of c/c adult mice (Fig. 2). We hypothesised that following DMM surgery, the increased ER stress experienced by chondrocytes as they upregulated collagen II (and therefore Tg^cog^) synthesis would increase either the rate of onset or the severity of disease in the *ColIITg*^*cog*^ mouse line. However, to our surprise, c/c mice displayed a chondroprotective effect at 2 weeks post-DMM. The chondroprotective effect observed in DMM-operated c/c mice was associated with a focal delay in apoptosis and with higher ambient levels of BiP protein in c/c chondrocytes prior to DMM (Fig. 3). Firstly, both ER stress and apoptosis were also involved in the early stages of mechanically induced cartilage thinning described in rat mandibular cartilage [31]. Secondly, the elevated levels of BiP protein in c/c mice were present in articular chondrocytes prior to DMM-induced OA (Fig. 3) even though at this time, the levels of *BiP* and *Tg*^*cog*^ mRNAs in cartilage were below the level of detection (Figs. 1 and 2, non-operated joints). The elevated levels of BiP protein in the c/c compared to +/+ chondrocytes, which presumably arises as a consequence of the exposure to increased ER stress during development and growth when the *Col2a1* promoter-driven *Tg*^*cog*^ expression was high [23], could explain why the *ColIITg*^*cog*^ mouse was partially protected and apoptosis delayed following DMM.

A higher ambient level of BiP induced by prior exposure to ER stress increases the efficiency with which the ER copes with a subsequent exposure to increased ER stress [32]. Furthermore, such pre-conditioning has previously been shown to be beneficial in OA [33–36]. Accordingly, the articular chondrocytes of the c/c compared to +/+ mice, were in a stronger position to deal with the increased ER stress associated with DMM possibly accounting for the lower levels of cartilage damage and the delayed apoptosis seen at 2 weeks (Figs. 2 and 3). The elevated levels of BiP protein in the *c/c* mice would also help suppress ER-derived oxidative stress resulting from the anabolic reaction in chondrocytes [37] indicated by the increased collagen II expression (Additional file 2: Figure S2). Together, these studies demonstrate the potential benefit of an adapted, pre-conditioned ER response, perhaps induced for instance by controlled heating and cooling of joints, in protecting against subsequent insults and thus delaying disease onset.

RNA-seq analysis revealed the most significantly dysregulated genes in +/+ DMM vs SHAM controls were also dysregulated in c/c DMM cartilage (Table 1) and in an independent microarray-based study [29]. The RNA for sequencing in the current study was collected from the whole medial tibial condyle rather than the tissue in the immediate vicinity of the OA lesion, and it is therefore not surprising that significant elevations in mRNAs encoding genes such as *BiP* and *Col2a1*, which at 2 weeks post-DMM were highly localised to the edge of the damaged cartilage (see Figs. 1 and 2), were not detected in +/+ DMM vs SHAM (Additional file 3: Table S2). More surprising was the absence of any significant increase in *BiP* mRNA in the cartilage of *ColIITg*^*cog*^ (c/c) DMM (Additional file 3: Table S3) despite the increased ambient BiP protein levels seen in the chondrocytes of the *ColIITg*^*cog*^ mice (Fig. 3 and Additional file 2: Figure S2). The increased BiP protein in the adult *ColIITg*^*cog*^ mouse chondrocytes are unlikely to be the consequence of short-term increased BiP synthesis but more likely arise from changes in protein turnover rates in the c/c mice [38].

Tg^cog^ expression is capable of activating all three arms of the UPR; IRE1, ATF6 and PERK [15, 39]. Moreover, in the current study, when looking for upstream regulators of genes dysregulated in c/c vs +/+ mice 2 weeks post-DMM, the *Xbp1*-driven UPR network was significantly activated in *ColIITg*^*cog*^ OA cartilage (Table 3), consistent with evidence of increased *Xbp1* splicing during cartilage development of *ColIITg*^*cog*^ mice [23]. These *Xbp1*-activated pathways downstream of the UPR sensor IRE1 can be considered a legacy of the *ColIITg*^*cog*^ chondrocytes having been conditioned to an elevated level of ER stress throughout development. XBP1 splicing is upregulated in human OA [19, 20], and it was also identified as an upstream regulator in mouse osteoarthritic tissue [29]. Additionally, the spliced active form of XBP1 is a negative regulator of apoptosis in OA [20]. Accordingly, DMM-operated c/c mice displayed a delay in chondrocyte apoptosis although our studies do not rule out the possibility that the delayed apoptosis is the result of less tissue damage rather than due to the increased BiP protein. Altogether, prior exposure to increased ER stress may have a chondroprotective role against the onset of OA perhaps through *Xbp1*-regulated pathways/networks together with the inherently higher ambient level of BiP protein present in the *ColIITg*^*cog*^ chondrocytes.

It is difficult to interpret the role of ER stress in disease progression in the *ColIITg*^*cog*^ mouse given that the chondrocytes start off with elevated levels of BiP. We therefore adopted a second approach by performing DMM on the *Atf6α*^*−/−*^ mouse, which has a compromised ability to respond to increases in ER stress in many tissues [25] including cartilage most evident through their severely compromised induction of BiP [30]. However, we were unable to detect any significant changes in OA severity in *Atf6α*^*−/−*^ mice following DMM suggesting that whilst increased ER stress is apparent early during disease onset in OA chondrocytes, control of the level of ER stress by ATF6α is not a major determinant of disease progression in DMM.

CHOP-knockout mice had reduced chondrocyte apoptosis and disease progression in a mechanically induced model of OA similar to DMM [22]. CHOP is a pro-apoptotic transcription factor that acts downstream of the PERK pathway under ER stress. Interestingly, PERK has emerged as a potential novel target for OA therapy [40]. However, ER stress is one of several forms of cellular stress that can promote apoptosis. Others include oxidative stress, osmotic stress, metabolic stress and heat shock [41], many of which have also been implicated in the OA disease mechanism(s) [42]. These later causes may be of more significance than ER stress in OA progression specifically given the lack of effect of the *ColIITg*^*cog*^ or *Atf6α*^*−/−*^ genotypes on OA severity at 4 and/or 8 weeks post-DMM.

It should be borne-in-mind that much of the work described and discussed above is based on studies using mechanically induced OA in genetically homogeneous lines, coupled with gene knockouts. However, when interpreting the relevance of these findings for the human OA condition, the genetic heterogeneity of the population together with the complex pattern of environmental influences must be taken into account.

## Conclusion

Our studies indicate that an increased capacity to effectively manage increases in ER stress in articular cartilage due either to genetic pre-disposition or pre-conditioning as a result of prior exposure to increased ER stress may be beneficial in delaying the onset of OA. Once significant tissue damage is established, ER stress does not play a significant role on disease progression.

## Additional files

Additional file 1: **Table S1.** Primer sequences for qPCR. (DOCX 18 kb)

Additional file 2: **Figure S1.** *ColIITg*^*cog*^mice have normal articular cartilage with no signs of degeneration. **Figure S2.** Increased BiP protein in articular cartilage of DMM-operated *ColIITg*^*cog*^ mice. **Figure S3.** Validation of RNAseq expression data by qPCR. Graphs displaying qPCR data relative to housekeeping β Actin (Act B) and normalised read counts from RNA-seq data of *FN1*, *MGP*, *SPP1, MMP3*, *BMP7 & Col2a1*, expression in articular cartilage from wild type (+/+) mice 2 weeks post DMM/SHAM and *ColIITg*^*cog*^ (c/c) mice 2 weeks post DMM. Each point represents an individual mouse (average of 2 technical replicates for qPCR data). Horizontal bars show the mean value for each gene. (DOCX 15459 kb)

Additional file 3: **Table S2.** Differentially regulated genes in +/+ DMM vs SHAM. **Table S3.** Differentially regulated genes in c/c DMM vs SHAM. **Table S4.** Differentially regulated genes in +/+ DMM vs c/c DMM. (XLS 276 kb)

Additional file 4: **Table S5.** Top uptream regulators identified in +/+ DMM mice compared to SHAM mice. (DOCX 52 kb)

## Data Availability

All data generated in this study is present as Additional files. Original RNA-seq data is available from ArrayExpress (E-MTAB-8266).

## Abbreviations

ATF6: Activating transcription factor 6
DMM: Destabilisation of the medial meniscus
ER: Endoplasmic reticulum
IHC: Immunohistochemistry
IPA: Ingenuity Pathway Analysis
IRE1: Inositol-requiring enzyme 1
OA: Osteoarthritis
OARSI: Osteoarthritis Research Society International
PERK: ER-kinase (PKR)-like ER kinase
Tg^cog^: cog mutant form of thyroglobulin
UPR: Unfolded protein response
