# Supplementary material for: Cartilage endoplasmic reticulum stress may influence the onset but not the progression of experimental osteoarthritis
Source: Arthritis Res Ther. 2019 Sep 11;21:206. doi: 10.1186/s13075-019-1988-6 (PMC6737683; doi:10.1186/s13075-019-1988-6)
Supplement: Supplementary file 1 — Table S1. Primer sequences for qPCR. (DOCX 18 kb) [file 13075_2019_1988_MOESM1_ESM.docx]

**Table S1: Primer sequences for qPCR**

| **Gene** | **Forward 5’ – 3’** | **Reverse 5’ – 3’** |
| --- | --- | --- |
| Act B | GGCTGTATTCCCCTCCATCG | CCAGTTGGTAACAATGCCATGT |
| Tg | AGGCATGTGCAGTGTGATGG | GGTACTGTGCTAGCACTGG |
| Hspa5 (BiP) | GGCACCTTCGATGTGTCTCTT | TCCATGACCCGCTGATCAA |
| Col2a1 | GAAGGATGGCTGCACGAAAC | CGGGAGGTCTTCTGTGATCG |
| Col6a3 | TTCACGGATGGAGCAGATGG | TAGCCACACGTTCAAGACCC |
| FN1 | CAATTCAGATTGCCTAGAAATACCT | CAACTGCATACAAAGTGTCTTCAA |
| MGP | AGAGAGTCCAGGAACGCAAC | AAGTAGCGGTTGTAGGCAGC |
| IL-11 | GGTGTTTGTCGCCTGGTC | AGGGGCAACGACTCTATCTG |
| SPP1 | GATGAACAGTATCCTGATGCCAC | TTGACTCATGGCTGCCCTTT |
| MMP3 | ATCCATGGAGCCAGGATTT | GACAGCATCCACCCTTGAGT |
| BMP7 | GAAGAAGTACAGAAACATGGTGGTC | CAAAGGTCAGGGTCTCAGGA |
