# Supplementary material for: Cartilage endoplasmic reticulum stress may influence the onset but not the progression of experimental osteoarthritis
Source: Arthritis Res Ther. 2019 Sep 11;21:206. doi: 10.1186/s13075-019-1988-6 (PMC6737683; doi:10.1186/s13075-019-1988-6)
Supplement: Supplementary file 2 — Figure S1. ColIITgcogmice have normal articular cartilage with no signs of degeneration. Figure S2. Increased BiP protein in articular cartilage of DMM-operated ColIITgcog mice. Figure S3. Validation of RNAseq expression data by qPCR. Graphs displaying qPCR data relative to housekeeping β Actin (Act B) and normalised read counts from RNA-seq data of FN1, MGP, SPP1, MMP3, BMP7 & Col2a1, expression in articular cartilage from wild type (+/+) mice 2 weeks post DMM/SHAM and ColIITgcog (c/c) mice 2 weeks post DMM. Each point represents an individual mouse (average of 2 technical replicates for qPCR data). Horizontal bars show the mean value for each gene. (DOCX 15459 kb) [file 13075_2019_1988_MOESM2_ESM.docx]

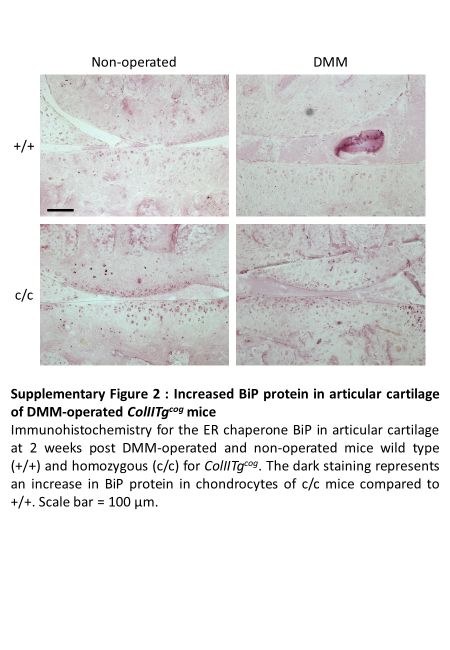


**Supplementary Figure 3: Validation of RNAseq expression data by qPCR**

Graphs displaying qPCR data relative to housekeeping β Actin (Act B) and normalised read counts from RNA-seq data of *FN1*, *MGP*, *SPP1, MMP3*, *BMP7 & Col2a1*, expression in articular cartilage from wild type (+/+) mice 2 weeks post DMM/SHAM and *ColIITg^cog^* (c/c) mice 2 weeks post DMM. Each point represents an individual mouse (average of 2 technical replicates for qPCR data). Horizontal bars show the mean value for each gene.
