# Supplementary material for: Cartilage endoplasmic reticulum stress may influence the onset but not the progression of experimental osteoarthritis
Source: Arthritis Res Ther. 2019 Sep 11;21:206. doi: 10.1186/s13075-019-1988-6 (PMC6737683; doi:10.1186/s13075-019-1988-6)
Supplement: Supplementary file 3 [file 13075_2019_1988_MOESM4_ESM.docx]

Supplementary Table 5: Top upstream regulators identified in +/+ DMM mice compared to SHAM mice

Upstream Regulators	Activation z-score	p-value of overlap	Target Molecules in Dataset
TGFB1	4.793	4.87E-39	ABCG1,ABCG2,Acan,ACKR2,ACVR1,ADAM17,ALDH18A1,ALOX12,AMD1,ANKH,ANXA11,AXL,BACE1,BGN,BHLHE40,BMP2,BMP6,BMP7,BTG1,BUB1B,CADM1,CAPNS1,CCND2,CD28,CD44,CDKN1A,COL6A1,COL6A2,COL6A3,COL8A1,COMP,CPQ,CST3,CTSH,CTTN,CYR61,DCN,DDIT4,DDR2,DKK3,DPYSL3,DSP,DTX4,ENPP1,ETS1,EXT1,F13A1,FBN1,FGF2,FN1,FOS,FURIN,FUT8,GALM,GAS1,GBP2,GDF6,GLI2,GNA13,GREM1,GSN,HBEGF,HMGA1,HSPG2,ID2,ID4,IFRD1,INHBA,KLF9,LAMB3,LOX,LTBP2,LTBP3,Ly6a (includes others),MGP,MMP3,MPHOSPH9,MPP6,MRPS6,MSN,MYB,MYO10,MYOF,NAMPT,NOG,NR4A2,NT5E,NUPR1,P4HA1,PAPPA,PDLIM5,PLAT,PLCB1,PMEPA1,PPP1R3C,PTGES,PTGS2,RAPGEF3,RBMS3,RHOC,RNF152,RSAD2,SBNO2,SCARB1,SCD,SELENBP1,SEMA7A,SERPINA3,SERPINE1,SGK1,SKIL,SLC1A2,SLC23A2,SLC39A14,SMC2,SMTN,SOCS3,SOD3,SOX9,SRC,TARDBP,TFRC,TG,TGFBI,TIMP1,TIMP3,TOP2A,TSC22D1,TUBB2A,USP25,VAT1,VCAM1,VCAN,VEGFA,VIM,ZFP36L2
EGF	4.295	7.39E-12	ABCG2,ADAM17,ALOX12,ARC,CCND2,CD44,CDKN1A,CXCL14,CYR61,DCN,ERRFI1,ETS1,FGF2,FN1,FOS,GAS1,HBEGF,ID2,INHBA,MMP3,NR4A2,ODC1,PLAT,PPAP2B,PTGES,PTGS2,SCD,SERPINA3,SERPINE1,SLC1A2,SLC4A7,SOCS3,ST3GAL3,TIMP1,TIMP3,VCAM1,VCAN,VEGFA,VIM,ZFP36L2
TGFB3	3.888	1.42E-11	BHLHE40,CDKN1A,CYR61,ETS1,FGF2,FN1,FOS,LTBP2,MGP,MMP3,MYO10,SCD,SERPINE1,SH3PXD2A,SKIL,TIMP3,TSPAN2,VEGFA
FGF2	3.802	4.90E-18	AKR1B1,BGN,CCND2,CDKN1A,CYR61,DCN,EFNB2,ENPP1,ENPP2,ERRFI1,ETS1,FBN1,FGF2,FN1,FOS,GREM1,HBEGF,INHBA,LECT1,LOX,MGP,MMP3,Nes,NOG,NPY,NR4A2,ODC1,PENK,PLAT,PTGS2,PTPRE,SCD,SERPINE1,SOCS3,SOX9,ST3GAL3,TIMP1,TIMP3,TOP2A,VCAM1,VEGFA,VIM
NUPR1	3.800	3.20E-04	ABL2,ADRB2,ATP6V0A1,BTG1,BUB1B,C3orf58,CYR61,DUSP8,ETS1,FAM120B,FGF1,FHL2,GBP2,HBEGF,HJURP,IRF2BP2,KANK2,NUPR1,RANBP6,RNF19B,SERPINE1,SLC6A6,TARDBP,ZFAND2A,ZFP36L1,ZSWIM6
PDGF BB	3.651	3.65E-18	ADRB2,ANXA11,BHLHE40,BMP6,C3,CD44,CDKN1A,CYR61,DCN,ENPP1,ETS1,FHL2,FN1,FOS,GBP2,GREM1,HBEGF,Hmga2,INHBA,KLHL21,MINOS1-NBL1/NBL1,MMP3,NAMPT,NPR3,NR4A2,ODC1,PLAT,PTGS2,SCD,SERPINA3,SERPINE1,SGK1,SLC6A6,SOCS3,SRSF7,TIMP1,TRA2B,VCAM1,VCAN,VEGFA,ZFP36L1
TNF	3.463	1.02E-16	ABCG2,Acan,ACKR2,ADAM17,ADRB2,AEBP1,AKR1B1,ALDH1A3,ANXA11,ARC,ATP1A1,BACE1,BGN,BHLHE40,BMP2,BTG1,BUB1B,C3,CCDC80,CCND2,CD28,CD44,CDKN1A,CLASP1,COL15A1,CTTN,CYP26B1,CYR61,DCN,EFNB2,EMP1,ENPP2,ETS1,EXT1,F11R,FGF2,FN1,FOS,GAB1,GBP2,GM2A,GPX4,HBEGF,HSD17B7,HSPG2,IDH2,IL18R1,INHBA,LAMB3,LGALS9B,LOX,LTBP2,MAP4K4,MGP,MMP3,NAMPT,NFKBIZ,NR4A2,NTN1,ODC1,OSMR,PAPPA,PENK,PIGR,PLAT,PPP1R3C,PTGES,PTGS2,RASD1,SCARB1,SCD,SCUBE1,SEMA3C,SERPINA3,SERPINE1,SGK1,SLC16A2,SLC1A2,SOCS3,SOD3,SOX9,ST3GAL3,TFRC,TG,THBS2,TIMP1,TIMP3,TM4SF1,TNN,UACA,VCAM1,VEGFA,VIM,YWHAG
MAPK14	3.364	5.52E-04	COL10A1,FN1,FOS,MMP3,PTGES,PTGS2,SGK1,SOCS3,TIMP1,TNN,TOP2A,VEGFA
ADCYAP1	3.324	2.32E-04	ARHGEF25,BMP6,ENPP2,FOS,GALNT7,H2AFZ,HK1,IDH2,LUM,NPR3,SCARB1,SERPINA3,SLC1A2,SOCS3,TGFBI,TSC22D1
IL6	3.324	7.08E-07	ABCG2,ACKR2,ACVR1,BGN,BMP2,BMP6,BUB1B,C3,CD44,CDKN1A,CST3,DCN,ENPP2,FN1,FOS,GBP2,ID2,LIG1,MMP3,MYB,NAMPT,NPY,PENK,PLAT,PTGES,PTGS2,SBNO2,SERPINA3,SERPINE1,SGK1,SLC39A14,SOCS3,SRC,TFRC,TIMP1,TOP2A,VCAM1,VEGFA,VIM
